# Cigarette Prices and Smoking Experimentation Among Zimbabwean Children: A Survival Analysis of the 2014 Global Youth Tobacco Survey

**DOI:** 10.1093/ntr/ntae048

**Published:** 2024-03-06

**Authors:** Chengetai Dare, Micheal Kofi Boachie, Corné van Walbeek

**Affiliations:** Research Unit on the Economics of Excisable Products, School of Economics, University of Cape Town, Rondebosch, South Africa; SAMRC/Wits Centre for Health Economics and Decision Science – PRICELESS SA, School of Public Health, University of Witwatersrand, Johannesburg, South Africa; SAMRC/Wits Centre for Health Economics and Decision Science – PRICELESS SA, School of Public Health, University of Witwatersrand, Johannesburg, South Africa; Research Unit on the Economics of Excisable Products, School of Economics, University of Cape Town, Rondebosch, South Africa

## Abstract

**Introduction:**

Zimbabwe has a smoking prevalence of 11.7% among the adult population (15 years and older). Thus, in the absence of effective tobacco control measures, the economic burden of tobacco use will be aggravated, especially considering the increasing tobacco industry activity in the country. Increasing cigarette prices is one possible strategy to reduce tobacco consumption. This study seeks to examine the relationship between cigarette prices and smoking experimentation among children in Zimbabwe, thereby expanding the evidence base for the likely impact of excise taxes on cigarette demand in low- and middle-income countries.

**Aims and Methods:**

A survival analysis using the Zimbabwe 2014 Global Youth Tobacco Survey data.

**Results:**

A 10% increase in the price of cigarettes reduces the probability of experimenting with smoking by 9%. Also, children are more likely to experiment with smoking if they have a smoking brother or father who smokes, or see teachers who smoke. The likelihood of experimenting with smoking is higher among boys than girls and is positively associated with age.

**Conclusions:**

There is strong evidence that increasing excise taxes can play an effective role in discouraging children from experimenting with cigarette smoking. Considering the relatively low excise tax burden in Zimbabwe, the government should consider substantially increasing the excise tax burden.

**Implications:**

With the number of smokers in low- and middle-income countries expected to increase as the industry intensively expands its market by targeting the youth, increasing excise taxes will play a significant role in preventing children from initiating smoking and help those who are already using tobacco to quit. An increase in the excise tax increases the retail price of tobacco products, making them less affordable, and reduces the demand for them.

## Introduction

There are over 1 billion smokers globally and about 80% of them live in low- and middle-income countries.^1,2^ Smoking is a major risk factor for cardiovascular and respiratory diseases, over 20 different types (or subtypes) of cancer, and many other chronic noncommunicable diseases.^3^ Globally, over 8 million people die every year from tobacco use and/or exposure to tobacco smoke.^2,4^ Although there has been a considerable decline in the number of smokers globally, the number of smokers is expected to increase in some countries.^5,6^ For instance, the number of smokers is projected to increase by 5.1%, from 59 million in 2015 to 62 million by 2025, in Sub-Saharan Africa,^5^ which is the second-largest percentage increase after the Eastern Mediterranean region.^7^ The expected increase in the number of smokers is attributed to increasingly aggressive tobacco industry marketing, with a substantial portion of these efforts targeted at the youth.^8^ With increasing tobacco industry activity in low- and middle-income countries, African countries are projected to progress to a tobacco epidemic by 2040.^8^

According to the World Health Organization’s Framework Convention on Tobacco Control (WHO FCTC), parties should work towards reducing the supply of and demand for tobacco. Zimbabwe ratified the FCTC in 2014, becoming the 181st party to the treaty. However, the decision to accept the FCTC does not appear to represent a softening of its historical opposition to the treaty.^9^ The government and the industry strongly promote the production of tobacco leaf while doing little to reduce the demand for tobacco.^9,10^ Currently, Zimbabwe is the largest producer of tobacco leaf in Africa and the seventh largest in the world.^11^ The smoking prevalence among the adult population (15 years and older) is estimated to be 11.7%, 21.8% among males, and 1.5% among females.^12^ Thus, in the absence of effective tobacco-control measures, the economic burden of tobacco use will be aggravated, especially considering the increasing tobacco industry activity in the country.^10^

Research has shown that most smokers start the habit early in life, usually before reaching 18 years of age,^6,13,14^ and that future smoking prevalence will be driven by experimentation among children.^13,14^ As the industry is intensively expanding its market by targeting the youth, countermeasures should be employed to prevent children from initiation and help those already using tobacco to quit.

Of the various tobacco-control measures, excise tax increases are an important strategy for reducing tobacco consumption.^15^ An increase in the excise tax increases the retail price of tobacco products, making them less affordable, and reduces the demand for them. The effectiveness of higher cigarette prices has been confirmed in several African countries.^6,7,15–20^ Children are more price-sensitive than adults, making tobacco taxation particularly effective with them.^20^

Data obtained from the Zimbabwe National Statistics Agency^21^ show that the average retail price for a packet of 20 cigarettes (of the most popular brands) ranged between US$0.61 and US$1.11 between 2005 and 2014, lower than the sub-Saharan African regional average of US$1.80, and the global average price of US$3.82.^22^ In 2020, taxes constituted 29.3% of the retail price,^22^ which is significantly below the 75% minimum target recommended by the World Health Organization.^23^ Due to the low prices, cigarettes in Zimbabwe are relatively more affordable than in other countries in the region. For the period 2010–2014, the affordability (measured as a percentage of GDP per capita needed to buy 2000 cigarettes) of the most-sold brand of cigarettes in Zimbabwe has been 7.3%, on average, compared to Zambia (9.6%), Mozambique (13.8%), and Malawi (55.1%).^24^

Although global knowledge about the effect of prices (or taxes) as a tobacco-control strategy is well established, there is, to the best of our knowledge, no evidence about the impact of prices on tobacco-use experimentation among children in Zimbabwe. This paper seeks to fill this knowledge gap by employing survival analysis to examine the effect of prices on the decision by children in Zimbabwe to experiment with cigarette smoking. The study follows the approach used by Dare et al.,^7^ Dauchy et al.,^17^ Asare et al.,^20^ Vellios and van Walbeek,^25^ and Boachie et al.^26^ in similar studies.

## Data and Methodology

Individual and household information on smoking behavior and other background characteristics was obtained from the Global Youth Tobacco Survey (GYTS).^27^ The survey was designed by the United States’ Centre for Disease Control and Prevention as a global standard tool for monitoring tobacco use among youth and guiding the implementation and evaluation of tobacco prevention and control programs.^27^ The GYTS is conducted using a two-stage cluster-sampling design, and does not track respondents over time but rather provides a snapshot of their smoking patterns.^26,28^ The GYTS has been valuable in studying smoking patterns in many countries, including those in Africa.

Zimbabwe has only one wave of the GYTS, conducted in 2014. The sample is nationally representative, covering a cross-section of junior students (Grade 7-Form 3). The dataset covers a sample of 6427 students, aged 12–17 years at the time of the survey. 1058 of the respondents provided insufficient information about their smoking status, reducing our sample size to 5369. 938 (17.5%) respondents reported being smoking experimenters or ever-smokers (defined as having smoked at least once or twice in their lifetimes).^7^ On average, smoking experimentation occurs at the age of 12. Summary statistics are shown in Table 1.

**Table 1.** Demographic Characteristics and Smoking Habits (%)

	*N* (and proportion of total sample)	Ever-smokers in the category
Students sampled (*N* = 5369)	*n* = 5369	*n* = 938 (17.5%)
Gender (*n* = 5329)		
Male	44.1	22.6
Female	55.9	12.8
Age (*n* = 5316)		
12	9.3	11.2
13	27.4	15.9
14	32.6	16.2
15	20.5	19.8
16	7.5	21.6
17	2.7	35.0
Father smokes (*n* = 5336)	17.6	31.1
Mother smokes (*n* = 5308)	12.6	38.6
Brother smokes (*n* = 5253)	11.5	41.1
Amount of pocket money per week (*n* = 5352):		
Usually do not have	30.6	14.9
Less than $1	35.8	12.5
$1–$2	17.9	15.7
$3–$5	8.2	27.7
$6–$8	1.7	30.8
$9–$10	2.2	44.4
Above $10	3.6	27.8
Sees teachers smoking in school buildings (*n* = 5194)	33.4	25.9
Regards quitting smoking as difficult (*n* = 5267)	51.7	16.9
Mean age (years)	14.0 (SD 1.18)	14.2 (SD 1.24)
Mean experimentation age (years)		11.6 (SD 2.78)

Transitions in smoking behavior are modeled in a duration framework, where the timing of the transition from having never smoked into smoking experimentation depends on the probability of experiencing a transition in period *t,* conditional on not having experienced a transition until period *t*; this is also known as the conditional failure rate or hazard rate.^7,17^ Smoking experimentation is defined as the first time the respondent smoked (at least part of) a cigarette.^7^ It is obtained from the question: “Have you ever tried or experimented with cigarette smoking, even one or two puffs?” However, cross-sectional data do not measure an individual’s transition into smoking experimentation, making it necessary to transform the cross-sectional GYTS data into pseudo-longitudinal data.^7,17,20,25^ The transformation allows for the duration (time-to-event) analysis.

As in previous studies,^7,20^ to obtain the pseudo-longitudinal data, we retroactively inferred the year of smoking experimentation using the GYTS question on the age of smoking experimentation: “How old were you when you first tried a cigarette?” For each age, the individuals were assigned a value of 0 if they did not experiment. The person is assigned a value of 1 in the year in which they tried a cigarette and then drops out of the dataset. The pseudo-longitudinal dataset was constructed on the assumption that individuals were exposed to the risk of experimenting with smoking at the age of eight.^7,20,25,29–31^ The oldest amongst the respondents was aged 17 when the survey was conducted in 2014. These respondents were aged eight in 2005. Therefore, those who started experimenting with cigarettes before 2005 are excluded from the dataset.

**Figure 1.**
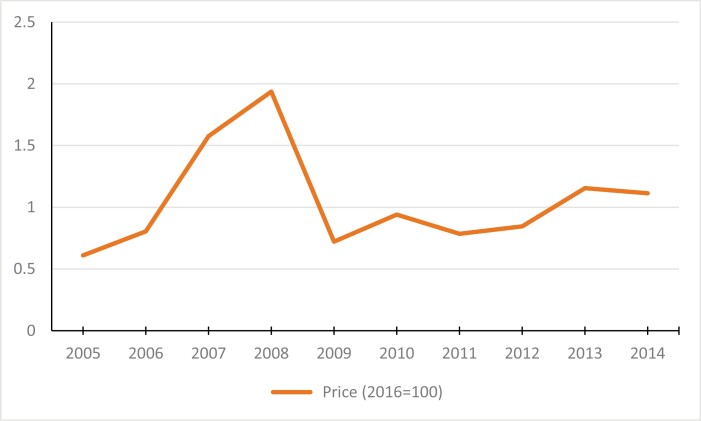
Average real price, 2005–2014.

The pseudo-longitudinal data is then merged with the national-level cigarette price, obtained from the Zimbabwe National Statistics Agency. This allows us to investigate the relationship between cigarette prices and smoking experimentation. The trends in retail price for a 20-pack of cigarettes are shown in Figure 1. The high prices in 2007 and 2008 are a reflection of the hyperinflation, recorded at 500 billion percent in mid-2008, which resulted in the abandonment of the Zimbabwean dollar in April 2009.^32^

As in Dare et al.,^7^ our baseline model for regression analysis uses a logistic distribution for the hazard function. This is because of its flexibility to allow non-monotone, time-dependent changes in the hazard rate.^7,17,33^ The regression model estimates the hazard of smoking experimentation for individual *i* in period *t* (*h*_it_) as a function of real cigarette prices (*price*_*t*_) and a vector of explanatory variables (**X**_i_), which include sociodemographics (age, gender, family members’ smoking status, income [pocket money] and respondents’ perceptions on quitting smoking).^7^ Gender takes the value of 1 for male and 0 for female. Smoking status of the respondent’s family members (father, mother, or brother) takes the value of 1 if they smoke, and 0 if do not smoke. The respondents’ income (pocket money) is derived from the GYTS question: “During an average week, how much money do you have that you can spend on yourself; however, you want?”^27^ The base is “I usually don’t have,” and the six other categories are “Less than $1,” “$1–$2,” “$3–$5,” “$6–$8,” “$9–$10,” and “Above $10.”

Respondents’ perceptions on quitting smoking are derived from the GYTS question: “Once someone has started smoking tobacco, do you think it would be difficult for them to quit?” The variable is coded as 1 for “Yes” and 0 for “No.”

The regression model is specified as:


$$\begin{array}{l} h_{it}=\mathrm{Pr}(experiment\;with\;smoking|no\;prior\;smoking) \end{array}$$

$$\begin{array}{l} =\beta_{1}price_{t}+\beta_{2}X_{it}+\varepsilon_{it}\; \end{array}$$ (1)

As in previous studies,^7,17^ we tested the robustness of the results by incorporating a discrete-time split population survival model, which relaxes the assumption that all individuals will eventually smoke. It first estimates each individual’s probability of ever experiencing a smoking transition, then weights the hazard function by this probability.^7,34^ The contribution of individual *i* to the log-likelihood function of smoking experimentation is:


$$\begin{array}{l} s_{i}=\ln\left\{\mathrm{Pr}(experiment\;\;\;with\;\;\;smoking)*f(t|t>0)\right\}+(1-s_{i})*\;\ln\left\{\begin{array}{l} \mathrm{Pr}(experiment\;\;\;with\;\;\;smoking)+\mathrm{Pr}(experiment\;\;\;with\;\;\;smoking)*\;f(t|t=0)\; \end{array}\right\} \end{array}$$ (2)

where *s*_*i*_ is a binary indicator for experimenting with smoking at some point during the period of observation, *t* is the time of experimentation measured in number of years since age eight, and *f*(*t*) is its probability density function.

We ran the split population survival model using Stata’s *spsurv* command, which uses a complementary log-log specification, and reports hazard ratios. The model has been used in numerous other studies.^7,25,34–36^ All analyses were done with Stata v.17.

### Patients and Public Involvement

Patients and the public were not involved in this study.

## Results

The results from the logistic and split population models are depicted in Table 2. The odds ratios (ORs) and hazard ratios are presented in exponentiated form and are broadly comparable. Although the hazard ratios are presented in the table, they are not discussed in this section.

**Table 2.** Regression Results: Logistic and Split Population Survival Models

	Logistic regression model		Split population model
	Coefficients	ORs	HRs
	(1)	(2)	(3)
Natural log of price	−0.878**	0.749**	0.761**
	(0.356)	(0.373)	(0.259)
Difficulty of quitting	0.189	1.208	1.078
	(0.193)	(0.234)	(0.106)
Father smokes	0.479**	1.614**	1.426***
	(0.231)	(0.400)	(0.178)
Mother smokes	0.315	1.370	1.371**
	(0.287)	(0.394)	(0.186)
Brother smokes	0.473**	1.605**	1.417***
	(0.261)	(0.432)	(0.184)
Teachers smoke in school buildings	0.419**	1.520**	1.733***
	(0.202)	(0.307)	(0.175)
Age	0.517***	1.677***	1.420***
	(0.048)	(0.081)	(0.040)
Male	0.403**	1.496**	2.432***
	(0.194)	(0.290)	(0.261)
Usually do not have pocket money	0.000	1.000	1.000
Less than $1	−0.096	0.909	1.136
	(0.272)	(0.247)	(0.158)
$1–$2	0.082	1.086	1.486***
	(0.300)	(0.326)	(0.228)
$3–$5	1.034***	2.811***	2.616***
	(0.306)	(0.860)	(0.452)
$6–$8	1.311***	3.708***	2.709***
	(0.481)	(1.782)	(0.743)
$9–$10	0.518	1.679	2.812***
	(0.381)	(0.639)	(0.653)
Above $10	−0.244	0.784	1.842***
	(0.378)	(0.296)	(0.396)
Constant	−3.879***	0.057***	0.387***
	(0.563)	(0.038)	(0.046)
Observations	38 036	38 036	38 036

Robust standard errors are in parentheses.

*** *p* < .01, ** *p* < .05, * *p* < .10.

The coefficient of the natural log of price is estimated to be −0.9 (95% CI: −1.6 to −0.1) (as shown in column 1), implying that a 1% increase in cigarette prices reduces the chance to experiment with smoking by 0.9%.

Children with a smoking father are more likely to have ever experimented with smoking a cigarette than those with a nonsmoking father (OR = 1.6, 95% CI: 0.9 to 2.8). Similarly, children with a smoking brother (sibling) are more likely to have ever smoked a cigarette than those with nonsmoking siblings (OR = 1.6, 95% CI: 0.9 to 2.7).

Those who see their teachers smoking inside school buildings are more likely to have ever smoked a cigarette than those who do not see teachers smoking inside school buildings (OR = 1.5, 95% CI: 1.0 to 2.3). As in Dare et al.,^7^ income (ie, having access to pocket money) did not have a clear effect. However, there is some suggestion that children who receive pocket money are more likely to experiment with smoking than children who do not receive pocket money.^37^

**Figure 2.**
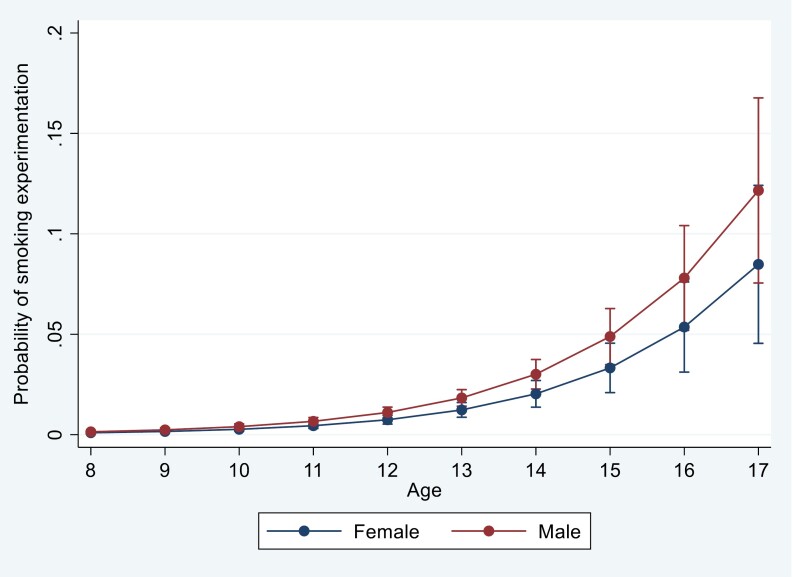
Hazard rates for smoking experimentation.

Boys are more likely to have ever experimented with smoking than girls (OR = 1.5, 95% CI: 1.0 to 2.2). The results also show that a 1-year increase in age increases the probability of experimenting with smoking (OR = 1.7, 95% CI:1.5 to 1.8). The smoking initiation hazard rates, by age and gender, are shown in Figure 2, and are derived from the regression analyses.

Figure 2 confirms that the probability of having ever experimented with smoking a cigarette is higher among males than among females and is positively correlated with age.

## Discussion

Given the lack of evidence on the link between cigarette price increases and smoking in Zimbabwe, this study investigated the relationship between price and smoking experimentation. The coefficient of the natural log of price is estimated to be − 0.9 in Zimbabwe. This implies that a 10% increase in cigarette prices would be associated with 9% reduction in the probability among Zimbabwean children aged 8–17 of experimenting with smoking.

This finding implies that increasing excise taxes could be an effective tobacco control tool in Zimbabwe. Holding all else constant, the increase in the excise tax raises the retail price of cigarettes, which consequently reduces the propensity to experiment with smoking.^6,17,20,38^ This finding is consistent with previous studies on this theme using a similar methodology.^7,17,20^

Our study finds that rising cigarette prices are associated with a lower likelihood of smoking experimentation among children. Compared to adults, children have lower incomes, and therefore higher prices put cigarettes out of reach of children. Considering that taxes constitute 29.3% of the retail price, which is significantly below the 75% minimum threshold recommended by the WHO, there is considerable room for the government to raise the excise tax on cigarettes further. Raising tobacco taxes generates revenue for government, while improving public health by discouraging tobacco use.^39^

Children learn a lot from their family members, and therefore pick up behaviors from siblings and parents. Children are inclined to practice what they see parents do. Also, parental smoking makes access to cigarettes easier as children could find cigarettes at home even if they had no money to buy them. Our findings resonate with the findings of Mays et al.,^40^ Gilman et al.,^41^ Jallow et al.,^42^ and Dare et al.,^7^ who found that children in smoking families were more likely to smoke than those in nonsmoking families. We also found that children are more likely to experiment with cigarette smoking if they see their teachers smoking inside school buildings. Because children spend a significant amount of their time in schools, teachers are considered as “second parents” and role models. This makes it easier for children to emulate any risky health behavior of teachers. Indeed, a recent survey in the United Kingdom showed that half of schoolchildren trusted their teachers more than parents and friends, indicating the crucial role teachers play in shaping behavior in society.^43^ Therefore, prohibiting smoking in and around schools would presumably reduce smoking among children. Considering this, the Zimbabwean government should come up with strategies that prohibit and/or discourage teachers from smoking in and around schools. The strategies may include educational campaigns against smoking in and around schools, or coming up with laws that prohibit such behavior by teachers. Educational campaigns can also be extended to parents, to discourage them from smoking in the presence of children.

Our results show that the probability of experimenting with smoking increases with age, which is in line with findings from the Gambia,^7^ Kenya,^17^ Ghana,^26^ and South Africa.^25^ As is the case in most other countries,^6,17,20,38^ smoking experimentation is lower among girls than among boys. This is reflected by the smoking prevalence in the general population, which is 21.8% among males and 1.5% among females.

## Limitation of the Study

There are some limitations to consider in interpreting the results of this study. The GYTS uses a self-reporting method to collect information from children. To the extent that individuals may not have an accurate recollection of past smoking events and dates on which such events occurred, this could make the dependent variable subject to measurement error with an unknown bias. However, considering that children usually have relatively short smoking records, the recall bias is likely to be limited.

Furthermore, several individual demographic and socioeconomic characteristics (eg, pocket money) were fixed at the time of the survey. Many of these socioeconomic variables (eg, amount of pocket money) could have changed in the years prior to the survey, which could have biased the results. The data covers a specific group of children (junior students), which may not be a good representation of children in Zimbabwe, including non-school-going children. Also, the dataset does not have information on respondents’ race and religion, which limits the analysis. However, considering that the Zimbabwean population is predominantly Black, it would be fair to assume that nearly all respondents are Black.

We did not control for smoke-free policy, which may lead to a measurement error. However, considering that teachers smoke in the presence of learners could be an indication that the smoke-free policy would be of insignificant effect in this study.

## Conclusion

We found the coefficient of the natural log of price to be −0.9, implying that a 10% rise in the price of cigarettes is associated with a 9% reduction in the probability of Zimbabwean children experimenting with smoking. This finding indicates that raising tobacco excise taxes can play an effective role in discouraging children from experimenting with smoking. Considering the current relatively low excise tax burden, the Zimbabwean government should consider increasing the excise tax burden on tobacco products in line with the recommendations of the World Health Organization.^23^

## Data Availability

Data are publicly available on: https://nccd.cdc.gov/GTSSDataSurveyResources/Ancillary/DataReports.aspx?CAID=2.
